# Performance of Liquid Biopsy‐Based Multi‐Omics Biomarkers for Early Detection of Gynecological Malignancies: A Prospective Study (PERCEIVE‐I)

**DOI:** 10.1002/advs.202401760

**Published:** 2025-04-03

**Authors:** Zheng Feng, Huijuan Ge, Jingshu Wang, Yanan Wang, Xiaoran Sun, Bo Yang, Siyu Cao, Chenlian Quan, Qinhao Guo, Yusheng Han, Feidie Duan, Fang liu, Jing Zhao, Guoqiang Wang, Yuzi Zhang, Shangli Cai, Xiaohua Wu, Hao Wen

**Affiliations:** ^1^ Department of Gynecologic Oncology Fudan University Shanghai Cancer Center Shanghai 200032 China; ^2^ Department of Oncology Shanghai Medical College Fudan University Shanghai 200032 China; ^3^ Department of Pathology Fudan University Shanghai Cancer Center Shanghai 200032 China; ^4^ Burning Rock Biotech Guangdong 510300 China

**Keywords:** cancer detection, gynecological malignancy, liquid biopsy, methylation

## Abstract

Liquid biopsy is a promising approach for early detection of gynecological malignancies. In the PERCEIVE‐I study, gynecological Cancer cases (*n* = 249) and age‐matched non‐cancer controls (*n* = 249) are randomly divided into training and test sets at a 1:1 ratio. Data derived from multi‐omics assays are obtained including a cell‐free DNA methylation panel targeting ≈490 000 CpG sites, a mutation panel comprising 168 genes, and eight tumor protein markers. The results showed that the methylation model outperformed the protein and mutation models, demonstrating higher sensitivity (77.2%) while maintaining similar specificity. The multi‐omics model combining methylation and protein markers achieved improved sensitivity (81.9%) with a good specificity (96.9%). The sensitivity varied across different stages, ranging from 66.7% to 100%. The model accurately identified the tissue of origin in 72.1% of cases. The superior performance of the methylation model highlights the potential of integrating multi‐omics for non‐invasive early detection of gynecological malignancies.

## Graphical abstract

Following a comparative analysis of omics‐based cancer detection models, a novel liquid biopsy‐based multi‐omics combined model is developed for the early detection of gynecological malignancies. By integrating cell‐free DNA methylation and tumor protein markers, the combined model demonstrates high specificity and sensitivity and is uniquely designed to address a spectrum of gynecological cancer types.

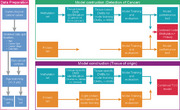

## 1 Introduction

Gynecological malignancies including cancers in the ovary, uterus, and cervix are among the notable diseases impacting women's lives nowadays.^[^ ^1^ ^]^ Early detection and diagnosis of these gynecological malignancies play a crucial role in improving their survival primarily because the 5‐year survival rates were favorable in early‐stage cancers compared with advanced gynecologic malignancies (80–90% vs 15–25%).^[^ ^2^, ^3^, ^4^ ^]^ Currently, effective population‐based screening methods in uterine and ovarian cancers do not exist, except that the thinprep cytologic test (TCT) is recommended for cervical cancer screening due to its capacity to reduce mortality rates and the relatively easy accessibility of the cervix.^[^ ^5^ ^]^ However, compliance with early screening for cervical cancer is less than 30% in China.^[^ ^6^ ^]^ Although risk‐reducing salpingo‐oophorectomy is recommended for patients at genetic high risk, ≈75% of ovarian cancers manifest as sporadic occurrences without certain risk factors.^[^ ^7^ ^]^ In addition, multiple examinations for one cancer type each time will inevitably result in time‐consuming procedures. Therefore, a paradigm shift from the traditional single‐cancer approach to a comprehensive screening encompassing all gynecological cancers can provide a more efficient solution for early detection of gynecological cancers.

Liquid biopsy is a promising approach for potential screening of gynecological cancers due to its advantages, such as convenient and minimally invasive sampling procedures, high compliance, and most importantly, the ability to simultaneously detect multiple types of cancer. Liquid biopsy for cancer screening involves the use of traditional tumor proteins as well as emerging biomarkers such as mutations and methylation. These biomarkers theoretically exhibit different performances in the early detection of gynecological cancers due to their distinct roles in carcinogenesis. Tumor proteins, such as CA125, Cancer Antigen 153 (CA153), Carbohydrate Antigen 19‐9 (CA19‐9), Carcinoembryonic Antigen (CEA), Ferritin (FERR), and Alpha‐Fetoprotein (AFP) and human epididymis protein 4 (HE4) for ovarian cancer and squamous cell carcinoma antigen (SCCA) for cervical cancer,^[^ ^8^ ^]^ often fail to identify early‐stage cancers and differentiate cancer from benign diseases, leading to moderate sensitivity and unsatisfied specificity. When it comes to circulating tumor DNA (ctDNA) mutation, detection of which relies on the limited amount of ctDNA released from tumor cells into the bloodstream. As a result, ctDNA mutation exhibits high specificity but relatively low sensitivity for early detection.^[^ ^9^ ^]^ In contrast, circulating cell‐free (cfDNA) methylation exhibits higher signal abundance than ctDNA mutation and possesses tissue specificity as a unique characteristic, which can be utilized for tracing tumor origin in multi‐cancer early screening.^[^ ^10^ ^]^ However, cfDNA methylation is also influenced by the quantity of ctDNA released into blood and the signal intensity can vary across different cancer types.

Recent studies have explored the performance of the above biomarkers, either individually or in combination, in early detection across multiple types of cancer. The OvaPrint classifier, which relied solely on cfDNA methylation, achieved 84.2% sensitivity and 96% specificity in differentiating high‐grade serous ovarian cancer from benign pelvic masses.^[^ ^11^ ^]^ The CCGA3 study, which focused on cfDNA methylation‐based detection of more than 50 cancer types, demonstrated a specificity of 99.5% with sensitivity of 83.1%, 80%, and 28% for cancers in the ovary, uterus, and cervix, respectively in the independent validation cohort.^[^ ^12^ ^]^ The accuracy of tissue of origin (TOO) for the three gynecological cancers was 70%, 35%, and 91%, respectively.^[^ ^12^ ^]^ Similarly, the THUNDER study developed a multi‐cancer early detection model targeting six cancers using the ELSA‐seq technique based on cfDNA methylation, and similar results were shown for ovarian cancer in terms of sensitivity and accuracy of TOO.^[^ ^13^ ^]^ In addition, the DETECT‐A study combined protein markers and ctDNA mutations, demonstrating a high specificity of 95.3% but a relatively low sensitivity of 27.5% in the population‐based prospectively intervention study.^[^ ^14^ ^]^ Specifically, among the six detected ovarian cancer samples, only one at stage I was detected, indicating limited sensitivity of proteins and ctDNA mutations in the early detection of gynecological malignancies. Furthermore, due to the suboptimal TOO, PET/CT examination became necessary in order to facilitate downstream clinical diagnosis. Altogether, these findings highlight the potential of liquid biopsy in the early detection of gynecological cancers, however, the complementary abilities of the above biomarkers in gynecological cancers and the development of rational integrated models to obtain a more suitable early detection strategy for gynecological cancers have not been fully explored.

Therefore, we conducted PERCEIVE‐I study (PERformance of multi‐cancer Early‐detectIon based on Various biomarkers in female cancers, NCT04903665) to explore the early detection performance of various biomarkers based on multi‐omics including cfDNA methylation, ctDNA mutation, and tumor proteins and to develop an effective early detection model for gynecological cancers.

## 2 Experimental Section

### 2.1 Study Design and Participants

**Figure 1:**
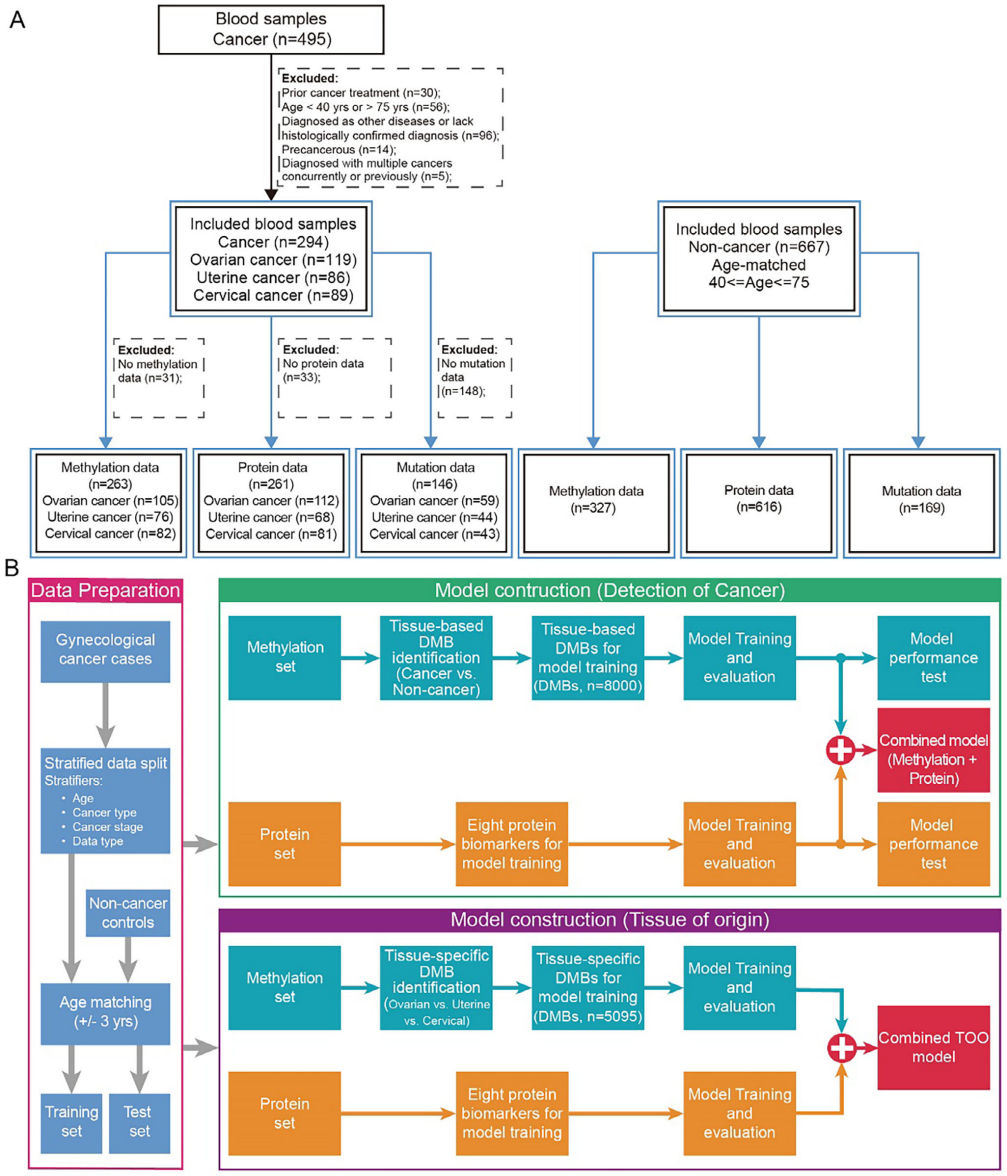
The flow chart of study design and model development. A) The overall description of study design. B) The diagram of data processing and model construction. TOO, tissue of origin; QC, quality control; DMB, differentially methylated blocks.

PERCEIVE‐I is a prospective study to explore gynecological malignancies early detection (GMED) models to simultaneously detect ovarian, uterine, and cervical cancers. Blood samples of patients with gynecological cancers were prospectively collected from the Fudan University Shanghai Cancer Center between February 2021 and October 2021. In addition, age‐matched noncancer samples were obtained from female individuals in another community‐based cohort (NCT04972201). All non‐cancer controls underwent low‐dose computed tomography (LDCT), abdominal ultrasound, mammography, blood tests, and urine tests, with no cancer incidence observed during the one‐year follow‐up. The inclusion and exclusion criteria for participants are detailed in Supporting Information and **Figure** **1**A.

The cancer samples were randomly divided into training and test sets at a ratio of 1:1 stratified by cancer type and age. Then noncancer samples were randomly age‐matched to the cancer samples in the training and test sets. During the matching process, a 1:1 ratio with an age difference of ±3 years was considered to ensure the comparability of age between cancer cases and noncancer controls in both training and test sets (Figure 1B). The primary outcomes were sensitivity, specificity, and TOO accuracy of cfDNA methylation, ctDNA mutations, and tumor protein markers in the early detection of gynecological cancers in the test set.

This study was approved by the Ethics Committee of Fudan University Shanghai Cancer Center (Approval number: 2012229‐13) and conducted according to the Helsinki Declaration. All participants provided written informed consent.

### 2.2 Sample Collection, Processing, and Sequencing

Blood samples were prospectively collected using Cell‐Free DNA BCT tubes (Streck, La Vista, NE). Additionally, formalin‐fixed paraffin‐embedded (FFPE) samples of cancer or adjacent were obtained and re‐reviewed by certified pathologists. The processing steps are detailed in Supplementary Materials. The detailed procedures for ELSA‐seq sequencing of cfDNA were consistent with previous descriptions.^[^ ^13^ ^]^ The testing results of serum tumor protein makers were collected from the medical records.

### 2.3 Identification of Cancer‐Specific and Tissue‐Specific Differentially Methylated blocks (DMBs)

The DNA methylation panel consists of 40 359 blocks, corresponding to ≈490 000 CpG sites. For the selection of cancer‐specific DMBs, the methylation levels of cancer and adjacent tissues were compared in ovarian, uterine, and cervical cancers, respectively. Blocks with a mean difference (meandiff) greater than 0.2 and an adjusted *p* less than 0.05 were identified as cancer‐specific DMBs for each cancer type. The DMBs from the three cancer types were combined, and then we utilized the Random Forest method to incrementally select a specified number of DMBs from a pool ranging from 500 to 15 000 features (increasing by 500 features each time) for model evaluation. During this process, we observed that the AUC value of the training dataset initially increased and then decreased. Consequently, we determined that the optimal feature set was achieved when 8000 DMBs were selected. As for the tissue‐specific DMBs, cancer and adjacent tissues of each cancer type were grouped together. Pairwise comparisons were performed between the three tissue types, and DMBs with a mean diff greater than 0.2 and an adjusted P less than 0.05 were defined as tissue‐specific DMBs. The DMBs from the three groups were then combined to construct the TOO model.

### 2.4 Construction of Single‐Omics Cancer Detection Model

The methylation model was built with the Support Vector Machine (SVM) algorithm and we employed grid search on the training dataset to fine‐tune the parameters of the SVM model. After extensive experimentation, we identified the optimal parameters: a regularization parameter C = 0.1 and a linear kernel function (kernel = “linear”). Based on these steps and the 8000 cancer‐specific DMBs, we applied five‐fold cross‐validation on the training dataset to develop five optimal models. These models were subsequently evaluated and validated on the test dataset. High specificity was the primary consideration during cutoff selection.

The protein model was constructed using eight serum tumor protein markers, namely, CA125, CA153, CA199, CEA, HE4, SCCA, FERR, and AFP. A highly stringent threshold was set for each protein marker to achieve relatively high specificity according to previous studies.^[^ ^14^ ^]^ A sample was reported to be positive when at least one protein marker value exceeded its corresponding threshold, which can be found in Supporting Information.

[Correction added on 14 April 2025 after online publication: protein construct design has been retained in the above paragraph.]

The cfDNA was sequenced by a next‐generation sequencing (NGS) panel targeting 168 cancer‐associated genes to detect gene mutations. A nonsynonymous mutation was considered positive if its allele frequency was higher than 0.1% for hot variants or 0.5% for nonhot variants. Similar to the protein model, a sample was considered positive with at least one positive nonsynonymous mutation.

### 2.5 Construction of Multi‐Omics Cancer Detection and Tissue‐of‐Origin Models

We evaluated the performance of the combined cancer detection model with a voting strategy on the intersection set of multi‐omics data. Within this dataset, we adopted a voting algorithm to construct the combined model. For each sample, the outcomes were evaluated by the respective single‐omics models independently. A sample was classified as positive in the combined model if it received a positive prediction from any one of the single‐omics models. (Figure 1B).

Tumor origin prediction can be formulated as a three‐class classification problem. The TOO model was constructed using the Catboost library based on the combined features of multi‐omics (Figure 1B). Fivefold cross‐validation was performed during the model training, resulting in five optimal models. For the model performance evaluation on the test set, only samples detected by the multi‐omics detection of cancer (DOC) model were included. The TOO model performance was primarily evaluated with the metrics of accuracy.

### 2.6 Statistical Analyses

All the analyses were conducted in R4.3.1 or Python 3.9.7. The two‐group comparisons of methylation were performed using t‐tests. The correction for multiple comparisons was conducted using the Benjamini‐Hochberg (BH) method. The machine learning algorithm was primarily implemented using the scikit‐learn and Catboost libraries. The confidence interval (CI) of proportions was calculated by the Clopper‐Pearson method. Heatmap was plotted using the R package of “pheatmap.” The nominal significance level was 5%, and CIs of 95% were 2‐sided unless otherwise specified.

## 3 Results

### 3.1 Characteristics of Participants

In this study, a total of 495 participants with gynecological cancers and 667 non‐cancer participants were included (Figure 1A). After careful evaluation of enrollment, quality control, and dataset partitioning, the training set included 244 participants (122 cancers and 122 non‐cancer controls), while the test set comprised 254 participants (127 cancers and 127 noncancer controls). Detailed information about these samples is provided in Table S1 (Supporting Information). As displayed in Table S1 (Supporting Information), age, cancer stage, and the proportion of each cancer type were generally balanced between the training and test sets (*p* > 0.05). All involved participants had the cfDNA methylation sequencing data and serum protein markers data. Among these participants, 120 individuals (65 cancers and 55 noncancer controls) in the training set and 122 individuals (66 cancers and 56 noncancer controls) in the test set had ctDNA mutation sequencing data, which was used for training and testing the ctDNA mutation model.

### 3.2 Identification of Cancer‐Specific and Tissue‐Specific Methylation Markers

**Figure 2:**
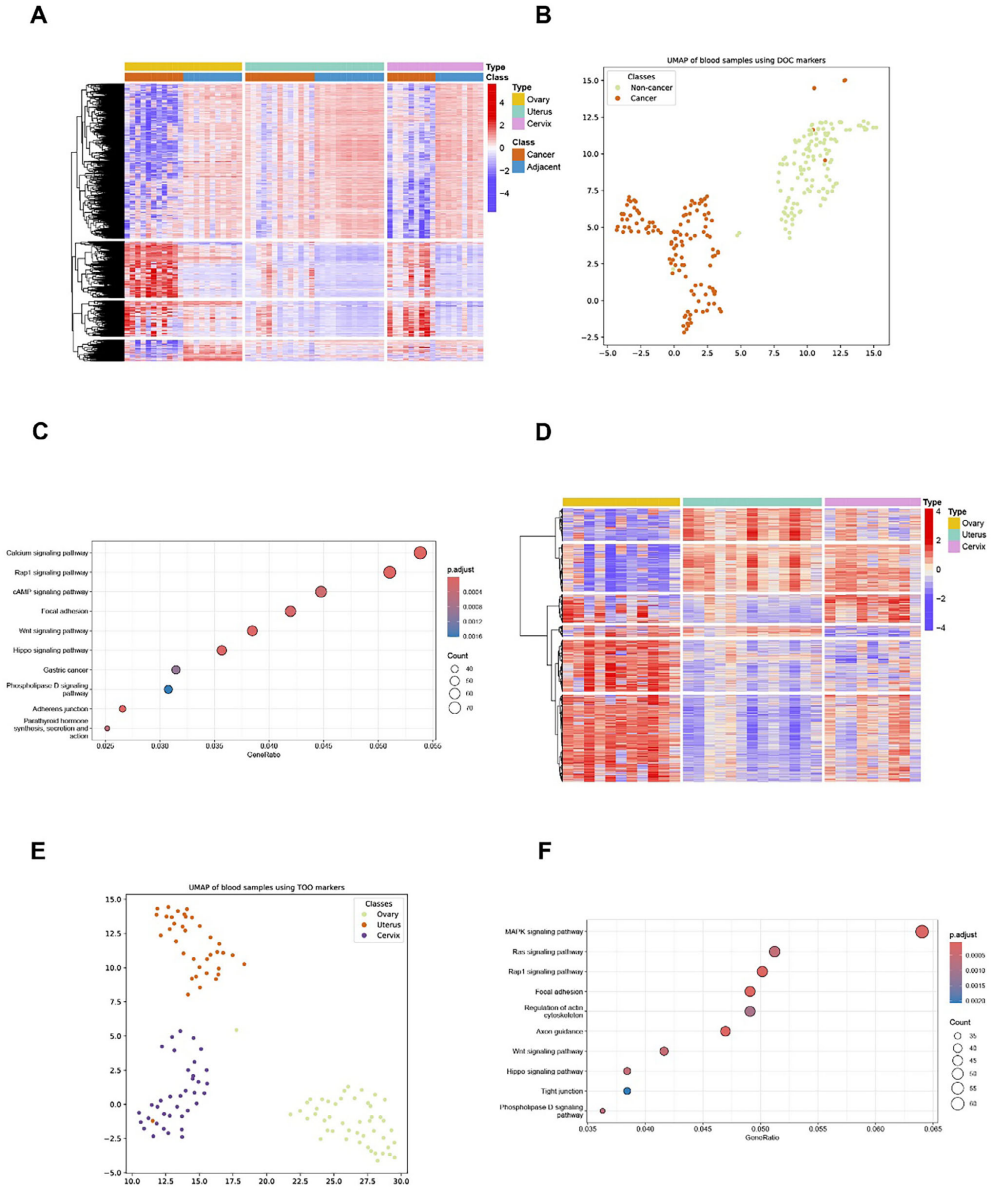
Selection of cfDNA methylation markers. A) The heatmap of DMBs between cancer and adjacent normal tissue for DOC; B) The UMAP based on the cancer‐specific DMB for DOC. C) The functional enrichment for DMBs of DOC. D) The heatmap of DMBs between various cancer/ adjacent tissues for TOO. E) The UMAP is based on the tissue‐specific DMBs for TOO. F) The functional enrichment for DMBs of TOO. DMB, differentially methylated block; DOC, detection of cancer; TOO, tissue of origin.

In the training set, 33 paired cancer and adjacent tissues from ovarian, uterine, and cervical cancers were employed to select cancer‐specific and tissue‐specific methylation markers. The characteristics of the above tissue samples are provided in Table S2 (Supporting Information). In total, 7225, 507, and 7189 cancer‐specific DMBs were discovered for cancers in the ovary, uterus, and cervix, respectively (Figure S1A–C, Supporting Information). After screening by random forest, we obtained the top 8000 cancer‐specific DMBs to be further included in the following model training (**Figure** **2**A). The 8000 DMBs were then tested with the blood samples in the divided training set, which showed a clear distinction between cancer and non‐cancer samples (Figure 2B). KEGG analysis revealed that these blocks were mainly associated with functions such as the Calcium signaling pathway, Rap1 signaling pathway, and cAMP signaling pathway (Figure 2C).

For tissue‐specific methylation markers, cancer and adjacent normal tissues within each cancer type were grouped together, and pairwise comparisons were conducted between different tissue types without matching based on age or cancer stage. Pairwise comparisons revealed 2606 tissue‐specific DMBs between ovarian and cervical tissues, 4080 DMBs between ovarian and uterine tissues, and 599 DMBs between uterine and cervical tissues (Figure S1D–F, Supporting Information). These tissue‐specific DMBs are depicted in Figure 2D, showing the distinct methylation patterns for different tissues. Subsequently, the DMBs were tested using blood samples from the segmented training set, revealing distinct methylation levels between ovarian, uterine, and cervical tissues (Figure 2E). KEGG analysis indicated that these DMBs were mainly associated with functional pathways such as the MAPK signaling pathway, Ras signaling pathway, and Rap1 signaling pathway (Figure 2F).

### 3.3 Performance of Single Omics Cancer Detection Models

**Figure 3:**
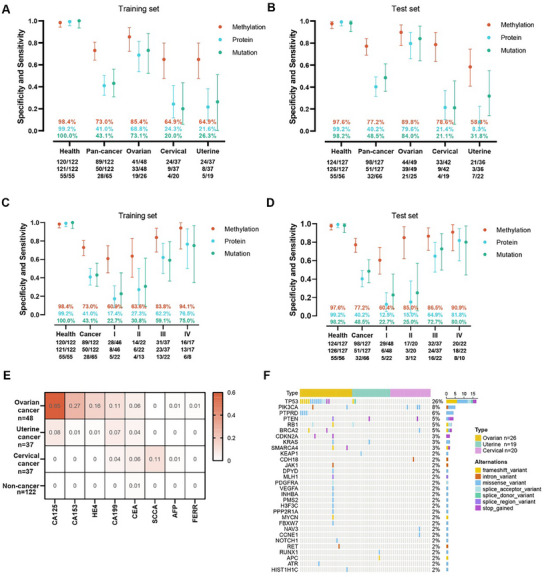
Comparison of three single omics DOC model. A) The overall sensitivity and sensitivity reported by cancer type of the three single omics DOC models in the training set, bars indicate 95% CI. B) The overall sensitivity and sensitivity reported by cancer type of the three single omics DOC models in the test set, bars indicate 95% CI. C) The overall sensitivity and sensitivity reported by the cancer stage of the three single omics DOC models in the training set, bars indicate 95% CI. D) The overall sensitivity and sensitivity reported by the cancer stage of the three single omics DOC model in the test set, bars indicate 95% CI. E) The heatmap of positive rates of each tumor protein across various cancers and health samples in the training set. F) The waterfall plot of ctDNA mutations across various cancer samples in the training set.

We developed three DOC models for each omics including methylation, protein, and mutation profiles in the training set and then tested in the test set. As depicted in **Figure** **3**A,B, the specificity of methylation DOC model achieved 98.4% (94.2–99.8%) and 97.6% (93.3–99.5%) in the training and test sets, with a sensitivity of 73.0% (64.2–80.6%) and 77.2% (68.9–84.1%), respectively. As for the protein DOC model, high specificity was also chosen. The specificity achieved 99.2% (95.5–100.0%) and 99.2% (95.7–100.0%) in the training set and test set, respectively. Correspondingly, the sensitivity in all cancers achieved 41.0% (32.2–50.3%) and 40.2% (31.6–49.2%), respectively, relatively lower than that of the cfDNA methylation model (training set: *p* < 0.001; test set: *p* < 0.001). In the mutation DOC model, the specificity achieved 100.0% (93.5–100.0%) and 98.2% (90.4–100.0%) in the training set and test set, respectively. Correspondingly, the sensitivity in all cancers achieved 43.1% (30.8–56.0%) and 48.5% (36.0–61.1%), respectively. All three DOC models showed the best predictive power in ovarian cancer [sensitivity of methylation DOC model: 85.4% (72.2–93.9%); sensitivity of protein DOC model: 68.8% (53.7‐81.3%); sensitivity of mutation DOC model: 73.1% (52.2–88.4%)] compared with the other two cancer types (Figure 3A). Similar results were also observed in the test set (Figure 3B).

Then the three DOC model's performance were assessed by disease stage (Figure 3C,D). In the test set of the methylation DOC model, the sensitivity increased with advanced stages and was 60.4% (45.3–74.2%), 85.0% (62.1–96.8%), 86.5% (71.2–95.5%), and 90.9% (70.8–98.9%) for patients with stage I, stage II, stage III and stage IV, respectively (Figure 3D). As for the protein DOC model, consistent with that in the methylation DOC model, the sensitivity increased with stages. In the mutation DOC model, a similar trend was observed (Figure 3C,D). The sensitivity stratified by cancer type and stage in three DOC models is provided in Table S3 (Supporting Information). Among the three DOC models, the methylation DOC model showed the best performance regardless of stage or cancer type, while the protein DOC model and mutation DOC model showed similar performance with relatively lower sensitivity.

We further illustrated the contribution of each tumor protein and gene mutation in detection of the gynecological cancers. In accordance with the literature,^[^ ^15^, ^16^ ^]^ the protein model displayed prominent detection capability for ovarian cancer. CA125, CA153, HE4, and CA199 showed significant contributions in identifying ovarian cancer when compared to the non‐cancer control group in the training set (CA125: *p* < 0.001; CA153: *p* < 0.001; HE4: *p* < 0.001; CA199: *p* = 0.002) (Figure 3E). Among these markers, CA125 exhibited the highest contribution, achieving a positive rate of 65%. In the case of uterine cancer, CA125, CA199, and CEA showed slight positivity. For cervical cancer, SCCA, CEA, and CA199 were found to be helpful in detection.

Regarding the selection of mutation markers, ovarian cancer exhibited a relatively higher mutation incidence of 73.1% (19/26) compared to the other two types of cancer in the training set (Figure 3F). TP53 mutation was the most common mutation in ovarian cancer, with 42.3% (11/26) cases of ovarian cancer patients carrying the simultaneous presence of two or more mutations. Interestingly, one ovarian cancer patient harbored 16 concurrent mutations. The mutation rates for uterine cancer and cervical cancer were 26.3% (5/19) and 20.0% (4/20), respectively.

### 3.4 Performance of Multi‐Omics Cancer Detection and Tissue‐of‐Origin Models

**Figure 4:**
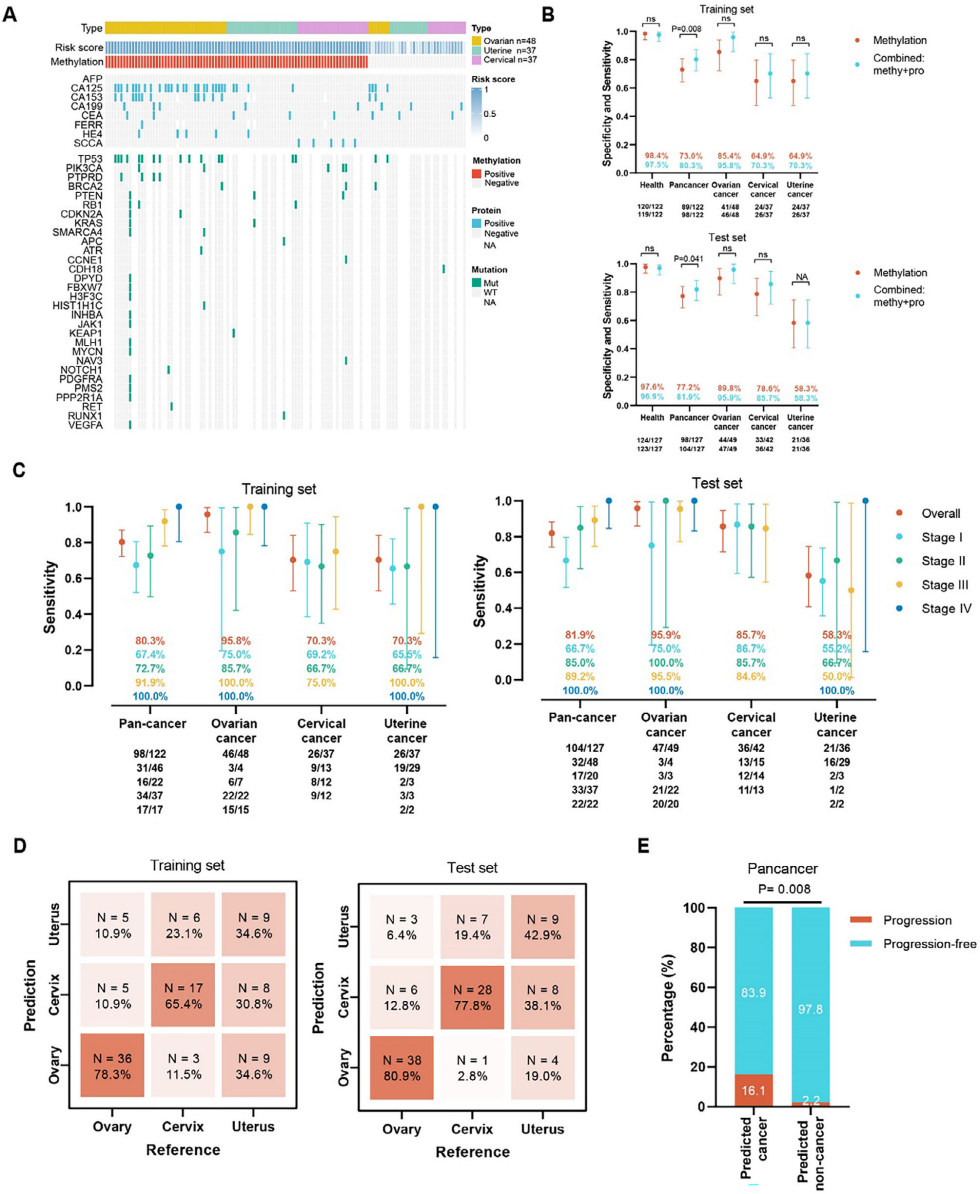
Performance of multi‐omics DOC and TOO model. A) Waterfall plot of three single omics DOC models across different cancer samples. B) The overall sensitivity and sensitivity stratified by cancer types of the methylation and multi‐omics DOC model in the training and test set, bars indicate 95% CI. C) The sensitivity of the multi‐omics DOC model by stage and cancer types, bars indicate 95% CI. D) Confusion matrix illustrating the accuracy TOO model in the training and test set. E) Bar chart depicting the correlation between the prediction of the multi‐omics DOC model and the progression of prognosis. DOC, detection of cancer; TOO, tissue of origin.

We then explored whether a multi‐omics combination would increase the sensitivity of detecting gynecological cancers. The contribution of three single omics DOC models to the detection of cancer cases at the individual level in the training set is depicted in **Figure** **4**A. Among 122 cancer cases, the methylation DOC model contributed 89 cases, and the protein DOC model contributed an additional nine cases which were missed by the methylation model. However, the mutation DOC model contributed only three more cases (**Figure** 4A), suggesting that the integration of ctDNA mutation information had a limited effect on model performance improvement. Thus, the methylation profile and protein profile were combined in the following development of an optimized multi‐omics DOC model.

Based on the above results, we further developed a multi‐omics early detection model named as GMED model combined methylation and protein profiles to further explore the improvement of model performance. Results showed that compared with the methylation DOC model, the integration of protein profile in the GMED DOC model obviously improved the overall sensitivity of cancer cases detection both in the training set [GMED model vs methylation model: 80.3% (72.2–87.0%) versus 73.0% (64.2–80.6%), P = 0.008] and test set [GMED model versus methylation model: 81.9% (74.1–88.2%) versus 77.2% (68.9–84.1%), P = 0.041] with similar specificity of 97.5% (93.0–99.5%) and 96.9% (92.1–99.1%), respectively (Figure 4B; Table S4, Supporting Information).

The sensitivity in patients with different cancer types and stages is depicted in Figure 4C. Results showed that the GMED DOC model had consistent performance between the training and test sets. In the test set, sensitivity increased with advanced stages and was 66.7% (51.6–79.6%), 85.0% (62.1–96.8%), 89.2% (74.6–97.0%), and 100.0% (84.6–100.0%) for all patients with stage I, stage II, stage III and stage IV, respectively. For different cancer types, compared with the single‐omics model, a performance improvement of the GMED model was observed in patients with ovarian cancer with an overall sensitivity of 95.9% (86.0–99.5%), and in patients with cervical cancer with an overall sensitivity of 85.7% (71.5–94.6%) (Figure 4C). As for subgroup analysis for the performance of the GMED DOC model, no significant difference was observed between different age groups [≥60 years vs < 60 years: 82.5% (71.9–89.1%) vs 81.6% (67.2–92.7%), *p* = 1.00], and different pathological types (ovarian cancer: high‐grade serous carcinoma versus others, *p* = 0.34; uterine cancer: endometrioid cancer versus others, *p* = 0.25; cervical cancer: squamous cell carcinoma versus nonsquamous cell carcinoma, *p* = 0.16) (Table S5, Supporting Information).

The GMED TOO model was further constructed by combining methylation and protein profiles. TOO was predicted in 80.3% (98/122) and 81.9% (104/127) of samples with cancer detected in the whole samples of the training and test set, respectively. Furthermore, samples identified by the GMED DOC model were counted for the GMED TOO, which was 63.3% (62/98) and 72.1% (75/104) in the training and test set, separately. In the training set, the accuracy of TOO was 78.3% (36/46), 34.6% (9/26), and 65.4% (17/26) for patients with ovarian cancer, uterine cancer, and cervical cancer, respectively (Figure 4D; Figure S2, Supporting Information). Similar results were observed in the test set that accuracy of TOO achieved 80.9% (38/47), 42.9% (9/21), and 77.8% (28/36) for patients with ovarian cancer, uterine cancer, and cervical cancer, respectively, indicating a promising utility to distinguish a specific cancer type from all the gynecological cancers (Figure 4D; Figure S2, Supporting Information).

In addition, we also explored the potential prognostic value of the GMED DOC model. As shown in Figure 4E, compared to the patients predicted as noncancer by the model, disease progression significantly occurred more often in the patients predicted as cancer (*p* = 0.008), suggesting that higher abnormal methylation and protein signals might be associated with disease prognosis in patients with gynecological cancers.

## 4 Discussion

In this study, we have explored different omics‐based cancer detection models and developed a liquid biopsy‐based multi‐omics model called GMED for the early detection of gynecological cancers by integrating cfDNA methylation and tumor protein markers. In the test set, the GMED model achieved an impressive specificity of 96.9% and sensitivity of 81.9%, as well as a TOO accuracy of 72.1%. To our knowledge, this signifies the pioneering utilization of a liquid biopsy‐based early detection model specifically tailored for multiple gynecological cancer types.

Even though gynecological cancers are among those diseases affecting women's lives, the incidence for each cancer is relatively low compared with lung cancer and breast cancer, making consecutively single‐cancer screening hardly an efficient and cost‐effective approach. Thus we developed the GMED model targeting multiple gynecological cancers simultaneously. A multi‐cancer detection model requires extremely high specificity to avoid unnecessary diagnostic procedures and high accuracy in locating cancers to facilitate diagnosis. In the present study, our GMED model had a specificity of 96.9% and TOO accuracy of 72.1% to minimize unnecessary diagnosis. Previously, CCGA series study developed a multi‐cancer early detection test covering more than 50 cancer types that encompassed the three gynecological cancers. However, in our present study, we mainly focused on gynecological cancers to increase the performance of multi‐cancer early detection in gynecological cancers When comparing our findings to their randomly assigned test set CCGA2^[^ ^17^ ^]^ and the independent validation cohort CCGA3,^[^ ^12^ ^]^ we observed a slightly lower specificity (96.9% vs 99.3% vs 99.5%), however, our model exhibited superior sensitivity for each cancer type (95.9% vs 70.6% vs 83.1% for ovarian cancer, 58.3% vs 25% vs 28% for uterine cancer, 85.7% vs 57.1% vs 80% for cervical cancer). Additionally, the GMED model achieved an overall sensitivity of 66.7% for stage I and 85% for stage II in the three gynecological cancers, and the respective sensitivity was 22.5% for stage I and 60% for stage II reported in CCGA3. Based on the incidence rates of ovarian cancer, uterine cancer, and cervical cancer from the 2020 Chinese Cancer Registry Annual Report,^[^ ^18^ ^]^ we further compared the incidence‐adjusted sensitivity of the GMED model with that of the CCGA3 model. The GMED model exhibited superior overall early sensitivity compared to CCGA3 (75.7% vs 45.4% for stage I and 83.7% vs 76.9% for stage II). Compared with the literature, commonly used clinical indicators for detecting asymptomatic ovarian cancer, such as CA125 and HE4, exhibit sensitivities no higher than 29% and 50% for stage I and II with specificity of 98%.^[^ ^19^ ^]^ In our study, GMED models achieved a sensitivity of 75% and 100% for stage I and II ovarian cancer. Regarding the TOO, the GMED model exhibited higher TOO in ovarian cancer and cervical cancer compared to CCGA3, with 80.9% versus 70.4% for ovarian cancer and 77.8% versus 35% for cervical cancer. However, in endometrial cancer, while the GMED model showed a relatively higher sensitivity of 58.3%, the TOO was only 42.9%. In contrast, CCGA3 reported a lower sensitivity of 28% for endometrial cancer but a higher TOO of 90.9%. Therefore, future efforts should focus on further exploring and improving the tissue of origin for endometrial cancer. Based on the above results, the GMED model demonstrates superior performance compared to previous research and can effectively meet the clinical demands for gynecological cancer screening.

Liquid biopsy encompasses various biomarkers, and in this study, we compared several widely studied biomarkers, including DNA methylation, proteins, and mutations. Our findings revealed that cfDNA methylation exhibited higher sensitivity (77.2%) while maintaining relatively high specificity (97.6%), surpassing the performance of proteins (40.2%) and mutations (48.5%). In the DETECT‐A study, a multi‐cancer early detection model combining mutations and proteins, 50% (3/6) of ovarian cancer and 50% (1/2) of uterine cancer exhibited TP53 mutations, aligning with the sensitivity of mutations in our study, ≈50%.^[^ ^14^ ^]^ Therefore, after a comprehensive comparison, cfDNA methylation, not ctDNA mutation, was chosen as the primary component of the early detection model. Similar results were also shown in CCGA1 study.^[^ ^20^ ^]^

Furthermore, we attempted to establish a combined model and found that the inclusion of proteins effectively improved overall sensitivity. The enhancement achieved by incorporating proteins primarily stems from their ability to complement the tumor‐released signals at the protein level, with performance being limited by the presence of sensitive protein markers for a particular tumor type. In ovarian and cervical cancers, the inclusion of proteins resulted in enhanced performance, with an additional 6–7% increase in sensitivity. Notably, CA125, HE4, and SCCA played pivotal roles in this improvement. However, although CA125 and CA199 showed slight contributions in uterine cancer, the sensitivity did not improve when combined with methylation, possibly due to that cases with abnormal protein levels showed dysfunctioned methylation patterns simultaneously. On the other hand, ctDNA mutation made a minimal contribution to the combined model of methylation and proteins. Among the 32 positive results predicted by the ctDNA mutation model, 94% (30/32) were also classified as positive in the cfDNA methylation and protein models. This may be due to the limited signals of cfDNA, particularly in early‐stage cancers.^[^ ^21^ ^]^ And cfDNA methylation exhibits abundant signals and synchronization with ctDNA mutations, thereby replacing the contribution of ctDNA mutations. When integrated with ctDNA mutation models, if the performance of the cfDNA methylation model performs exceptionally well, the additional contribution of mutation panels is diluted. Consequently, employing panels that encompass an excessive number of genes may be less cost‐effective. Additionally, cfDNA methylation not only encompasses tumor‐specific signals but also includes tissue‐related signals, providing more comprehensive information for early cancer detection.^[^ ^20^ ^]^ Therefore, in the future, a combination of methylation and proteins may be an ideal approach.

Although we developed the first pan‐cancer early detection model specifically targeting gynecological tumors and conducted a systematic comparison of the performance of different omics‐based DOC models in this study, there are also some limitations to our study. First, we did not include enough samples from individuals with severe benign diseases, which might have led to an overestimation of the model's specificity. Second, further improvements in marker selection and model construction are still needed to enhance TOO performance. In our study, the protein models were not directly constructed using modeling techniques but instead utilized cutoff values. This approach may underestimate the performance of the proteins. Third, the lack of an independent validation cohort and the limited statistical power (due to sample size constraints) may explain the nonsignificant differences in methylation patterns and GMED performance when detecting specific cancer types. To address these limitations, future studies should utilize multi‐center, large‐scale clinical cohorts to refine the model and prospectively validate its generalizability. The ongoing PERCEIVE‐II study (NCT: 06001099) aims to address this.

In summary, our study shows that cfDNA methylation markers outperform tumor protein markers and ctDNA mutations in detecting gynecological malignancies, particularly in the early stages. The GMED model for the early detection of multiple gynecological cancers by incorporating cfDNA methylation and tumor protein markers holds promise as an effective and convenient method in clinical use. Optimization of the model and prospective validation are necessary in the future to ensure its reliability and accuracy.

## Conflict of Interest

The authors declare no conflict of interest.

## Supporting information



Supporting Information

## Data Availability

The data that support the findings of this study are available from the corresponding author upon reasonable request.
