# Supplementary material for: Performance of Liquid Biopsy‐Based Multi‐Omics Biomarkers for Early Detection of Gynecological Malignancies: A Prospective Study (PERCEIVE‐I)
Source: Adv Sci (Weinh). 2025 Apr 3;12(20):2401760. doi: 10.1002/advs.202401760 (PMC12120774; doi:10.1002/advs.202401760)
Supplement: Supplementary file 1 — Supporting Information [file ADVS-12-2401760-s001.docx]

**Supplementary Materials**

Title: Performance of Liquid Biopsy-based Multi-Omics Biomarkers for Early Detection of Gynecological Malignancies: A Prospective Study (PERCEIVE-I)

Supplement to: Zheng Feng, Huijuan Ge, Jingshu Wang, Yanan Wang, Xiaoran Sun, Bo Yang4, Siyu Cao, Chenlian Quan, Qinhao Guo, Yusheng Han, Feidie Duan, Fang liu, Jing Zhao4, Guoqiang Wang, Yuzi Zhang, Shangli Cai, Xiaohua Wu, Hao Wen.

Supplementary Methods 2

Study design and participants 2

Inclusion and exclusion criteria for the training and the test sets 3

Sample collection, processing and sequencing 3

Cutoff selection of the methylation model 4

Thresholds of protein markers for cancer detection model 5

Enrichment analysis of key pathways 5

Prognostic analysis 5

Supplementary Figures 5

Supplementary Tables 9

# Supplementary Methods

## Study design and participants

PERCEIVE-I study is a prospective study initiated by Fudan University Shanghai Cancer Center to detect gynecological malignancies early detection including ovarian, uterine and cervical cancers.

Fudan University Shanghai Cancer Center had received approval from its institutional review board and was qualified to enroll participants and to implement the PERCEIVE-I study compliant with good clinical practice (GCP) guidelines. Clinical information, demographics (age, sex, race, etc.), medical history, family history, social history, medication use, smoking and alcohol history, and medical data relevant to cancer status were collected on all participants at baseline (time of bio-specimen collection). All cancer cases must have a pathological confirmed diagnosis. Clinical stage was assigned according to the FIGO Staging Manual (2018 version of ovarian and cervical cancer, 2009 version of endometrial cancer).

For included cancer blood samples (n=294), those with no data of a single omics was excluded. Therefore, 14 samples without methylation detection and 17 samples failing in the lab quality control process were excluded from the methylation dataset. Samples with incomplete protein markers (n=33) were removed, and 148 samples with insufficient volume for mutation testing were also omitted prior to matching.

The non-cancer controls analyzed in this study were recruited from a community-based cohort study (NCT04972201), the cancer status who were confirmed through transvaginal ultrasonography (TVUS), thinprep cytologic test (TCT), low-dose computed tomography (LDCT), abdominal ultrasound, mammography, blood tests, and urine tests. Additionally, a 1-year telephone follow-up was conducted to confirm their absence of cancer. Among these non-cancer participants, approximately half were diagnosed with benign conditions such as uterine fibroids/adenomyosis, ovarian cysts, and cervical cysts/polyps/fibroids, as determined by imaging assessments (55 cases in the training set and 64 cases in the test set).

## Inclusion and exclusion criteria for the training and the test sets

Inclusion criteria for cancer participants include:

- Participants were female over 18 years old;
- Participants were able to provide blood samples;
- A confirmed pathological diagnosis of any stage (I‒IV) of ovarian cancer, endometrial cancer and cervical cancer;
- Before blood collection, the subjects did not receive any of the following anti-tumor treatment: tumor surgery for any purpose, local or systemic radiotherapy and chemotherapy, targeted therapy (including anti-angiogenesis), immunotherapy, Cancer vaccine, hormone therapy.

Exclusion criteria for cancer participants include:

- Participants who were pregnant or lactating;
- Recipient of organ transplant or prior non-autologous (allogeneic) bone marrow or stem cell transplant;
- Recipient of blood transfusion within 7 days prior to blood draw;
- Individuals who received or were undergoing curative cancer treatment within three years prior to blood draw;
- Participants who took medication with anti-tumor effects within 30 days prior to blood draw;
- Participants with concurrent or heterozygous for other malignant tumors or multiple primary tumors.

## Sample collection, processing and sequencing

Blood samples were prospectively collected using Cell-Free DNA BCT tubes (Streck, La Vista, NE). After proper labelling, these samples were transported to the laboratory within 72 hours while maintaining a temperature range of 15-35°C. Plasma cfDNA was extracted from the blood samples using the QIAamp Circulating Nucleic Acid Kit (Qiagen, Germantown, MD) and the QIAsymphony® Circulating DNA Kit (Qiagen). Additionally, cancerous or benign/adjacent formalin-fixed paraffin-embedded (FFPE) samples were obtained and reevaluated by certified pathologists. The DNA from the FFPE samples was extracted using the QIAamp DNA FFPE Tissue Kit (Qiagen) following the manufacturer's protocol. The quality and quantity of DNA were assessed using the Qubit dsDNA HS Assay (Thermo Scientific, Waltham, MA) and the LabChip GXII touch 24 (PerkinElmer, Waltham, MA), respectively. The extracted cfDNA and tissue DNA were stored in elution buffer at -80°C. For the analysis of cfDNA, a custom targeted methylation analysis was performed using ELSA-seq, with a minimum of 8 ng of plasma cfDNA samples used for the analysis. In cases where sufficient cfDNA was available, OncoCompass Target analysis of 168 cancer-related genes was conducted. The levels of tumor protein markers in the serum samples were documented in the in-house testing report.

## Cutoff selection of the methylation model

Given that the model is used for early cancer detection, a high false-positive rate would subject many participants to unnecessary follow-up tests and cause significant psychological stress. Therefore, high specificity (low false-positive rate) is particularly crucial for early detection models. Consequently, the cutoff value was selected to maximize specificity while maintaining an acceptable sensitivity level. In this process, we examined several potential cutoff values.

The cutoff corresponding to the maximum Youden's Index, which balances sensitivity and specificity. The cutoffs corresponding to specificities of 0.9, 0.95, and 0.99. Ultimately, we chose the cutoff value that achieved a specificity of 0.984 while maintaining a sensitivity of 0.73. This cutoff is closest to the target specificity of 0.95, indicating a high level of specificity without compromising the overall model performance. This choice ensures that our model remains robust in early detection while minimizing the burden of false positives on patients.

## Thresholds of protein markers for cancer detection model

Highly stringent threshold was set for each protein marker to achieve a relatively high specificity. Expressly, the thresholds for AFP, CA125, CA153, CA199, and CEA were set to 21.321, 557, 98, 92, and 7.507, which were the same as thresholds used in the study of DETECT-A. For FERR, HE4, and SCCA, thresholds were set to 3000, 1320, and 15, respectively, which were 10 or 20 times of their upper limits. In the application stage, a sample will be reported positive when having at least one protein biomarker value exceeding its corresponding threshold.

## Enrichment analysis of key pathways

Kyoto Encyclopedia of Genes and Genomes (KEGG) enrichment analysis was conducted to determine whether a series of a priori-defined biological pathways were enriched by “clusterProfiler” R package with the genes corresponding to the cancer-specific and tissue-specific methylation blocks. The top 10 significantly enriched pathways were shown and arranged in the order of their gene ratio.

## Prognostic analysis

The prognostic value of gynecological malignancies early detection (GMED) model was explored in the 245 patients with available follow-up data. The median follow-up time was 27.7 months, and disease progression occurred in 33 patients. The difference of disease progression between patients predicted as cancer and non-cancer by GMED model was compared by Fisher’s exact test.

# Supplementary Figures

**Figure S1**. **Selection of** **differentially methylated blocks.** **(A)** The volcano plot of differentially methylated blocks between ovarian cancer and adjacent tissue for DOC. **(B)** The volcano plot of differentially methylated blocks between uterine cancer and adjacent tissue for DOC. **(C)** The volcano plot of differentially methylated blocks between cervical cancer and adjacent tissue for DOC. **(D)** The volcano plot of differentially methylated blocks between ovarian and cervical cancer/ adjacent tissues for TOO. **(E)** The volcano plot of differentially methylated blocks between ovarian and uterine cancer/ adjacent tissues for TOO. **(F)** The volcano plot of differentially methylated blocks between cervical and uterine cancer/ adjacent tissues for TOO. DOC, detection of cancer; TOO, tissue of origin.


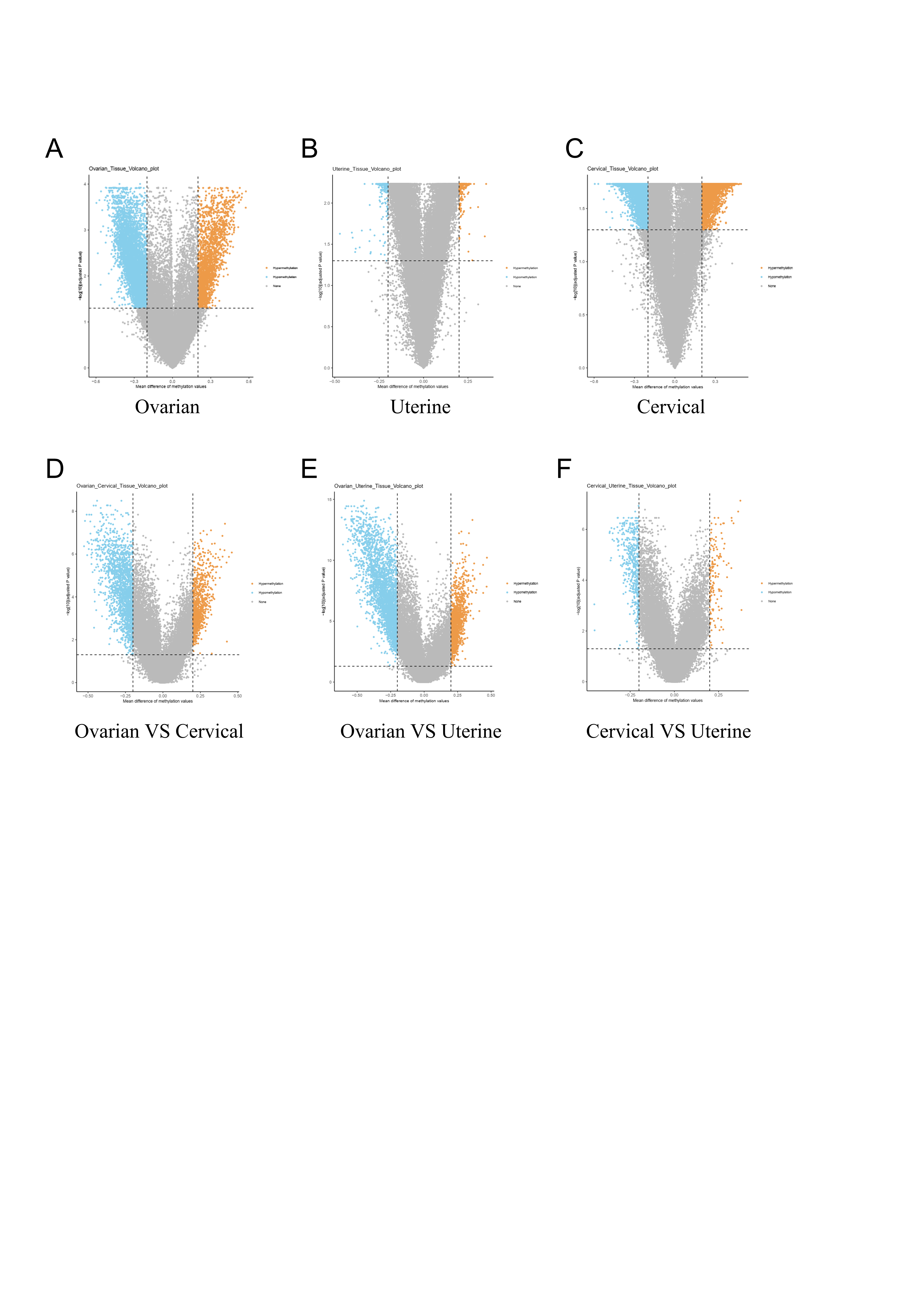


**Figure S2**. **Accuracy of tissue of origin by cancer type in the training and test sets.**

#
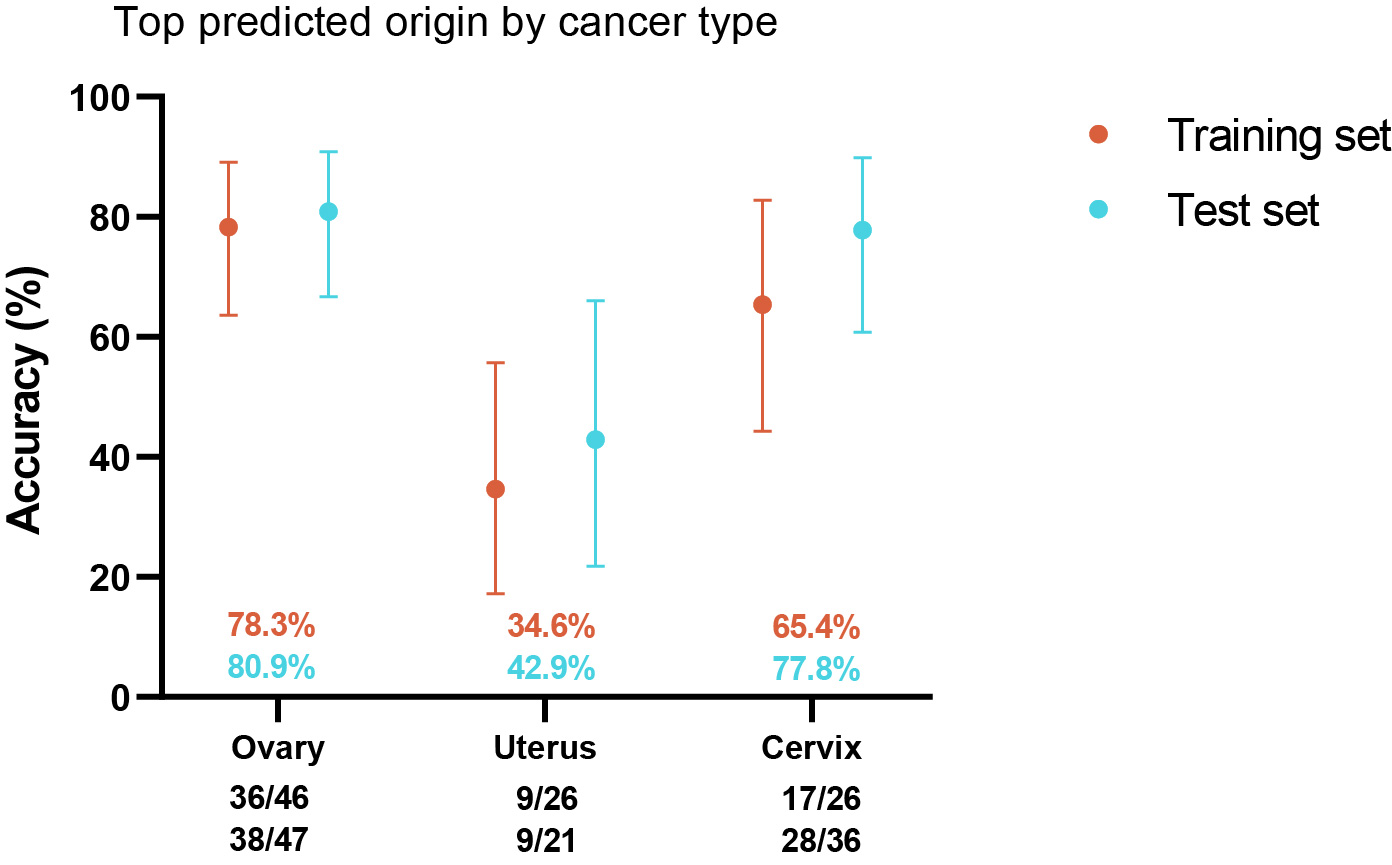
Supplementary Tables

**Table S1. Baseline characteristics of the participants** **included in the study.**

|  | **Training set** | |  | | **Test set** | |  | |  | |  |
| --- | --- | --- | --- | --- | --- | --- | --- | --- | --- | --- | --- |
|  | **Cancer  (n = 122)** | **Non-cancer  (n = 122)** | |  | | **Cancer  (n = 127)** | **Non-cancer  (n = 127)** |  | | **P value** | |
| **Age, median (range)** | 56 (41–75) | 56 (40–74) | |  | | 56 (41–74) | 56 (40–74) |  | | 0.88 | |
| **Cancer type, n (%)** |  |  | |  | |  |  |  | | 0.89 | |
| Ovarian cancer | 48 (39.3%) |  | |  | | 49 (38.6%) |  |  | |  | |
| Uterine cancer | 37 (30.3%) |  | |  | | 36 (28.3%) |  |  | |  | |
| Cervical cancer | 37 (30.3%) |  | |  | | 42 (33.1%) |  |  | |  | |
| **Stage, n (%)** |  |  | |  | |  |  |  | | 0.88 | |
| I | 46 (37.7%) |  | |  | | 48 (37.8%) |  |  | |  | |
| II | 22 (18.0%) |  | |  | | 20 (15.7%) |  |  | |  | |
| III | 37 (30.3%) |  | |  | | 37 (29.1%) |  |  | |  | |
| IV | 17 (13.9%) |  | |  | | 22 (17.3%) |  |  | |  | |
| Gynecological benign conditions, n(%) |  | 55 (45.1%) | |  | |  | 64 (50.4%) |  | |  | |

**Table S2. Baseline characteristics of the participants with gynecological cancer in the tissue DNA methylation marker selection set.**

|  | **Marker selection set** |
| --- | --- |
|  | **Cancer/adjacent**  **(n = 33)** |
| **Age, median (range)** | 56 (52–61) |
| **Cancer type, n (%)** |  |
| Ovarian cancer | 11 (33.3%) |
| Uterine cancer | 13 (39.4%) |
| Cervical cancer | 9 (27.3%) |
| **Stage, n (%)** |  |
| I | 15 (45.5%) |
| II | 8 (24.2%) |
| III | 6 (18.2%) |
| IV | 4 (12.1%) |

**Table S3. Sensitivity and Specificity of single omics DOC models by cancer type and stage.**

|  |  |  | **Training set** |  |  |  |  | **Test set** |  |
| --- | --- | --- | --- | --- | --- | --- | --- | --- | --- |
|  | **Specificity** |  | **Sensitivity** |  |  | **Specificity** |  | **Sensitivity** |  |
| **Cancer type** |  | **Ovarian cancer** | **Uterine cancer** | **Cervical cancer** |  |  | **Ovarian cancer** | **Uterine cancer** | **Cervical cancer** |
| **Methylation** |  |  |  |  |  |  |  |  |  |
| Total | 98.4% (94.2–99.8%)  (120/122) | 85.4% (72.2–93.9%) (41/48) | 64.9% (47.5–79.8%) (24/37) | 64.9% (47.5–79.8%) (24/37) |  | 97.6% (93.3–99.5%)  (124/127) | 89.8% (77.8–96.6%) (44/49) | 58.3% (40.8–74.5%) (21/36) | 78.6% (63.2–89.7%) (33/42) |
| Stage I |  | 75.0% (19.4–99.4%) (3/4) | 58.6% (38.9–76.5%) (17/29) | 61.5% (31.6–86.1%) (8/13) |  |  | 75.0% (19.4–99.4%) (3/4) | 55.2% (35.7–73.6%) (16/29) | 66.7% (38.4–88.2%) (10/15) |
| Stage II |  | 57.1% (18.4–90.1%) (4/7) | 66.7% (9.4–99.2%) (2/3) | 66.7% (34.9–90.1%) (8/12) |  |  | 100% (29.2–100%) (3/3) | 66.7% (9.4–99.2%) (2/3) | 85.7% (57.2–98.2%) (12/14) |
| Stage III |  | 90.9% (70.8–98.9%) (20/22) | 100% (29.2–100%) (3/3) | 66.7% (34.9–90.1%) (8/12) |  |  | 90.9% (70.8–98.9%) (20/22) | 50.0% (1.3–98.7%) (1/2) | 84.6% (54.6–98.1%) (11/13) |
| Stage IV |  | 93.3% (68.1–99.8%) (14/15) | 100% (15.8–100%) (2/2) | NA |  |  | 90.0% (68.3–98.8%) (18/20) | 100% (15.8–100%) (2/2) | NA |
| **Protein** |  |  |  |  |  |  |  |  |  |
| Total | 99.2% (95.5–100%)  (121/122) | 68.8% (53.7–81.3%) (33/48) | 21.6% (9.8–38.2%) (8/37) | 24.3% (11.8–41.2%) (9/37) |  | 99.2% (95.7–100%) (126/127) | 79.6% (65.7–89.8%) (39/49) | 8.3% (1.8–22.5%) (3/36) | 21.4% (10.3–36.8%) (9/42) |
| Stage I |  | 50.0% (6.8–93.2%) (2/4) | 17.2% (5.8–35.8%) (5/29) | 7.7% (0.2–36.0%) (1/13) |  |  | 50.0% (6.8–93.2%) (2/4) | 0% (0–11.9%)  (0/29) | 26.7% (7.8–55.1%) (4/15) |
| Stage II |  | 57.1% (18.4–90.1%) (4/7) | 0% (0–70.8%)  (0/3) | 16.7% (2.1–48.4%) (2/12) |  |  | 66.7% (9.4–99.2%) (2/3) | 0% (0–70.8%)  (0/3) | 7.1% (0.2–33.9%) (1/14) |
| Stage III |  | 72.7% (49.8–89.3%) (16/22) | 33.3% (0.8–90.6%) (1/3) | 50.0% (21.1–78.9%) (6/12) |  |  | 86.4% (65.1–97.1%) (19/22) | 50.0% (1.3–98.7%) (1/2) | 30.8% (9.1–61.4%) (4/13) |
| Stage IV |  | 73.3% (44.9–92.2%) (11/15) | 100% (15.8–100%) (2/2) | NA |  |  | 80.0% (56.3–94.3%) (16/20) | 100% (15.8–100%) (2/2) | NA |
| **Mutation** |  |  |  |  |  |  |  |  |  |
| Total | 100% (93.5–100%)  (55/55) | 73.1% (52.2–88.4%) (19/26) | 26.3% (0–51.2%) (5/19) | 20.0% (5.7–43.7%) (4/20) |  | 98.2% (90.5–100%)  (55/56) | 84.0% (63.9–95.5%) (21/25) | 31.8% (13.9–54.9%) (7/22) | 21.1% (6.1–45.6%) (4/19) |
| Stage I |  | 100% (15.8–100%) (2/2) | 20.0% (4.3–48.1%) (3/15) | 0% (0–52.2%)  (0/5) |  |  | NA | 23.5% (6.8–49.9%) (4/17) | 20.0% (0.5–71.6%) (1/5) |
| Stage II |  | 25.0% (0.6–80.6%) (1/4) | 0% (0–97.5%)  (0/1) | 37.5% (8.5–75.5%) (3/8) |  |  | 100% (15.8–100%) (2/2) | 50.0% (1.3–98.7%) (1/2) | 0% (0–36.9%)  (0/8) |
| Stage III |  | 76.9% (46.2–95.0%) (10/13) | 100% (15.8–100%) (2/2) | 7.7% (0.2–36.0%) (1/7) |  |  | 85.7% (57.2–98.2%) (12/14) | 50.0% (1.3–98.7%) (1/2) | 50.0% (11.8–88.2%) (3/6) |
| Stage IV |  | 85.7% (42.1–99.6%) (6/7) | 0% (0–97.5%)  (0/1) | NA |  |  | 77.8% (40.0–97.2%) (7/9) | 100% (2.5–100%) (1/1) | NA |

**Table S4. Comparison of GMED DOC model and methylation DOC model performance by cancer subtype.**

|  |  | **Group** | | | |
| --- | --- | --- | --- | --- | --- |
|  | **Health** | **Pan-cancer** | **Ovarian cancer** | **Uterine cancer** | **Cervical cancer** |
| **Training set** |  |  |  |  |  |
| Methylation model | 98.4%(120/122) | 73% (89/122) | 85.4% (41/48) | 64.9% (24/37) | 64.9% (24/37) |
| GMED model | 97.5%(119/122) | 80.3% (98/122) | 95.8% (46/48) | 70.3% (26/37) | 70.3% (26/37) |
| P value | 1.000 | 0.008 | 0.074 | 0.480 | 0.480 |
| **Test set** |  |  |  |  |  |
| Methylation model | 97.6%(124/127) | 77.2%(98/127) | 89.8%(44/49) | 78.6%(33/42) | 58.3%(21/36) |
| GMED model | 96.9%(123/127) | 81.9%(104/127) | 95.9%(47/49) | 85.7%(36/42) | 58.3%(21/36) |
| P value | 1.000 | 0.041 | 0.248 | 0.248 | NA |

**Table S5. Sensitivity of GMED DOC model by age and pathological subtype.**

|  |  | **Test set** | | | |  |  |
| --- | --- | --- | --- | --- | --- | --- | --- |
| **Cancer type** |  | **Pan-cancer** | **Ovarian cancer** | **Uterine cancer** | **Cervical cancer** |  | **P value** |
| **Age** |  |  |  |  |  |  | 1.00 |
| < 60yrs |  | 81.6% (71/87) | 100% (32/32) | 54.2% (13/24) | 83.9% (26/31) |  |  |
| ≥ 60yrs |  | 82.5% (33/40) | 88.2% (15/17) | 66.7% (8/12) | 90.9% (10/11) |  |  |
| **Ovarian cancer subtype** |  |  |  |  |  |  | 0.34 |
| High-grade serous carcinoma |  |  | 97.4% (38/39) |  |  |  |  |
| Others |  |  | 88.9% (8/9) |  |  |  |  |
| **Uterine cancer subtype** |  |  |  |  |  |  | 0.25 |
| Endometrioid cancer |  |  |  | 51.9% (14/27) |  |  |  |
| Others |  |  |  | 77.8% (7/9) |  |  |  |
| **Cervical cancer subtype** |  |  |  |  |  |  | 0.16 |
| Squamous cell carcinoma |  |  |  |  | 91.3% (21/23) |  |  |
| Non-squamous cell carcinoma |  |  |  |  | 78.9% (15/19) |  |  |
